# Programmed Cell Death 1 (PD-1) Inhibitors in Renal Transplant Patients with Advanced Cancer: A Double-Edged Sword?

**DOI:** 10.3390/ijms20092194

**Published:** 2019-05-03

**Authors:** Hung-Chih Lai, Ji-Fan Lin, Thomas I.S. Hwang, Ya-Fang Liu, An-Hang Yang, Chung-Kuan Wu

**Affiliations:** 1 Division of Hematology and Oncology, Department of Internal Medicine, Shin-Kong Wu Ho-Su Memorial Hospital, Taipei 111 Taiwan; ctpetlai@gmail.com; 2 Precision Medicine Center, Department of Teaching and Research, Shin-Kong Wu Ho-Su Memorial Hospital, Taipei 111, Taiwan; jifanlin@hotmail.com (J.-F.L.); A001133@ms.skh.org.tw (Y.-F.L.); 3 Division of Urology, Department of Surgery, Shin-Kong Wu Ho-Su Memorial Hospital, Taipei 111, Taiwan; thomashwang0820@gmail.com; 4 School of Medicine, Fu-Jen Catholic University, New Taipei 242, Taiwan; 5 Department of Pathology and Laboratory Medicine, Taipei Veterans General Hospital, Taipei 112, Taiwan; ahyang@vghtpe.gov.tw; 6 Division of Nephrology, Department of Internal Medicine, Shin-Kong Wu Ho-Su Memorial Hospital, Taipei 111, Taiwan

**Keywords:** graft rejection, immunotherapy, PD-1 inhibitor, renal transplant

## Abstract

Given advancements in cancer immunity, cancer treatment has gained breakthrough developments. Immune checkpoint inhibitors, such as programmed cell death 1 (PD-1) inhibitors, are the most promising drugs in the field and have been approved to treat various types of cancer, such as metastatic melanoma, head and neck squamous cell carcinoma, and urothelial carcinoma. However, whether PD-1 inhibitors should be administered to renal transplant patients with advanced cancer remains unclear because the T-cells produced after administration of these inhibitors act against not only tumor antigens but also donor alloantigens. Thus, the use of PD-1 inhibitors in kidney-transplanted patients with advanced cancer is limited on account of the high risk of graft failure due to acute rejection. Hence, finding optimal treatment regimens to enhance the tumor-specific T-cell response and decrease T-cell-mediated alloreactivity after administration of a PD-1 inhibitor is necessary. Thus far, no recommendations for the use of PD-1 inhibitors to treat cancer in renal transplant patients are yet available, and very few cases reporting kidney-transplanted patients treated with PD-1 inhibitors are available in the literature. Therefore, in this work, we review the published cases and suggest feasible approaches for renal transplant patients with advanced malignancy treated by a PD-1 inhibitor. Of the 22 cases we obtained, four patients maintained intact grafts without tumor progression after treatment with a PD-1 inhibitor. Among these patients, one maintained steroid dose before initiation of anti-PD1, two received immunosuppressive regimens with low-dose steroid and calcineurin inhibitor (CNI)-elimination with sirolimus before initiation of anti-PD-1 therapy, and one received combined anti-PD-1, anti-vascular endothelial growth factor (VEGF), and chemotherapy with unchanged immunosuppressive regimens. mammalian target of rapamycin (mTOR) inhibitors and anti-VEGF may act as regulators of tumor-specific and allogenic T-cells. However, more studies are necessary to explore the optimal therapy and ensure the safety and efficacy of PD-1 inhibitors in kidney-transplanted patients.

## 1. Introduction

The development of immunosuppressive drugs is the key to suppressing allograft rejection. In the past two decades, increased immunosuppressive efficiency significantly reduced the incidence of acute rejection. With increased immunosuppression, however, there is also an increased rate of post-transplant infections and malignancies. The risk of cancer after transplantation is increased by three- to five-fold compared with that of the general population, and the prognosis of transplanted patients with malignancy is poorer than that of other cancer patients [1,2]. The high risk of cancer after transplantation has been linked to environmental carcinogenic risk factors, the comorbidities of transplanted recipients, and the detrimental effects of immunosuppressants such as activation of oncogenic viruses, carcinogenic effects of the medications, and loss of immunity for immune-surveillance [3]. The prognosis of recipients diagnosed with cancer is worse than that for cancer patients in the general population; therefore, cancer-related death in post-kidney transplantation is common and requires heightened surveillance [4]. It has been demonstrated that immunosuppressants can influence the efficacy of cancer treatment and lead to poorer tolerated to oncologic treatments [3]. Therefore, interactions between immunosuppressants and cancer therapies should be taken into account when formulating a therapeutic strategy.

**Figure 1:**
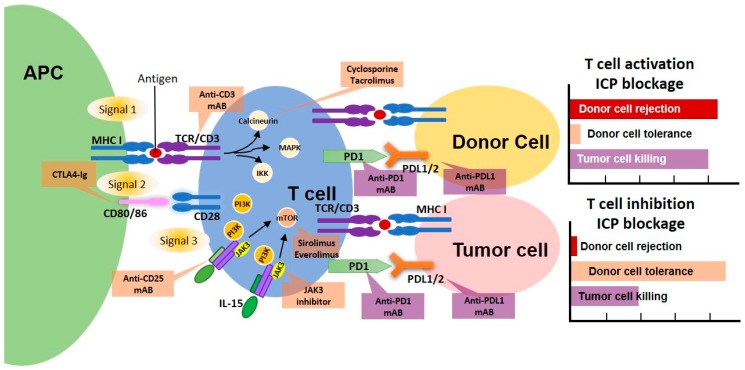
The role of T-cell suppression and immune checkpoint blockage in tumor and organ rejection. Activation of a T cell via 3-signal pathway by an antigen presenting cell (APC) is illustrated. The donor cells are rejected when the T cells are activated. Therefore, the immunosuppressants, which inhibit CD3 (Anti-CD3 mAB), calcineurin (Cyclosporine or Tacrolimus), CD80/86 (CTLA4-Ig), IL-2 signaling (Anti-CD25 mAB), JAK3 (JAK3 inhibitor), mammalian target of rapamycin (mTOR) (Sirolimus or Everolimus), and those interfere with the proliferative phase in the cell cycle (MPA, mycophenolate mofetil (MMF), azathioprine, and Fk778; not illustrated) are the key to successful post-transplantation outcomes. On the other hand, the employment of immune checkpoint inhibitors in transplant patients with cancer may increase the tumor killing while giving the chance for graft rejection. Therefore, fine-tuning the immunosuppressants and immune checkpoint inhibitors in transplanted patients with cancer is vital in achieving graft tolerance while treating cancer. APC, antigen presenting cell; CTLA4, cytotoxic T-lymphocyte-associated protein 4; IL-2, interleukin-2; IL-15, interleukin-15; JAK3, Janus kinase 3; PI3K, phosphoinositide 3-kinase; TCR, T-cell receptor; MHC I, major histocompatibility complex; mTOR, mammalian target of rapamycin; PD1, programmed cell death 1; PDL 1/2, programmed death-ligand 1/3.

Given advancements in cancer therapy, development of immune checkpoint inhibitors employing antibodies targeting programmed cell death 1 (PD-1), PD-1 ligand (PD-L1), or monoclonal antibodies against cytotoxic T-lymphocyte-associated antigen 4 (CTLA-4) in patients with various types of cancer has steadily increased. However, options for immune checkpoint inhibitors are limited in organ transplant patients because of the high risk of graft rejection and immunosuppression due to chronic use of this treatment (Figure 1). As such, reviewing the efficacy and safety of immune checkpoint inhibitors in organ transplant patients with cancer is necessary. Here, we focus on the efficacy and safety of PD-1 inhibitors in renal transplant patients, given that these drugs may be influenced by different tissue types or immune factors in other organ transplant recipients. Moreover, no data regarding the use of PD-L1 inhibitors, such as atezolizumab, avelumab, or durvalumab, in renal transplant patients are available. Finally, evidence suggests that anti-CTLA4 monoclonal antibodies are associated with a lower risk of rejection in renal transplant recipients compared with anti-PD-1 monoclonal antibodies [5,6,7]. By reviewing the usage of PD-1 inhibitors in renal transplant recipients with advanced cancer, we attempted to provide possible factors that influence the efficacy and safety of these inhibitors.

## 2. PD-1 Inhibitors in Renal Transplant Patients with Cancer

Tumor cells are known for their ability to inhibit T-cell-mediated immunosurveillance and the effector response by upregulating inhibitory checkpoint molecules, such as the programmed death ligands PD-L1 and PD-L2, which interact with PD-1 on T-cells to suppress their activation [8]. Hence, anti-PD-1 monoclonal antibodies that block PD-1 molecules can promote T-cell activation, consequently stimulating the cell-mediated and humoral anti-tumor response. PD-1 inhibitors such as nivolumab and pembrolizumab have been approved to treat metastatic melanoma, metastatic non-small cell lung cancer, renal cell carcinoma, classical Hodgkin’s lymphoma, head and neck squamous cell carcinoma (SCC), urothelial carcinoma, hepatoma previously treated with sorafenib, and metastatic gastric or gastroesophageal junction adenocarcinoma [9]. Renal transplant recipients are at higher risk of developing skin cancers [10], urologic malignancies [11], and other malignancies than the general population [12]. Therefore, knowledge of the anti-tumor effects of PD-1 inhibitors is undoubtedly necessary for renal transplant patients with the aforementioned cancers. Unfortunately, activation of T-cells by PD-1 inhibitors is not specifically against malignant cells; activated T-cells also attack donor alloantigens in transplanted kidneys. In addition, the PD-1/PD-L1 axis is critical to inducing and maintaining the peripheral allograft tolerance in transplant recipients. For example, this axis is involved in the induction of regulatory T-cells (Tregs), which play an important role in suppressing T-cell activation after exposure to alloantigens in renal transplant recipients [13]. Thus, activated-T cells against donor-alloantigen in the transplanted kidney, or the attenuated function of Tregs via blocking the PD-1, can result in graft rejection. However, not all renal transplant patients developed graft rejection after receiving PD-1 inhibitors. Allograft characteristics may be one of the pivotal influential factors. For example, PD-L1 has been identified to protect against alloreactive T-cell-mediated injury in renal tubular epithelial cells. Increased levels of PD-L1 on the graft kidneys possibly promote the host T-cell suppression by preventing the activation of alloreactive T-cells and stimulating apoptosis [14]. Therefore, high levels of PD-L1 in donor tissue can prevent pathologic alloreactivity and graft failure [15]. Aside from the allograft, other factors can affect graft survival after PD-1 inhibitor treatment. Considering these issues, developing an optimal means to maximize the therapeutic effects and minimize the toxicity of PD-1 inhibitors is an important endeavor.

## 3. Graft Failure After Administration of a PD-1 Inhibitor in Renal Transplant Patients with Advanced Cancer

Our data on the efficacy and safety of PD-1 inhibitors in kidney-transplanted patients with advanced malignancies mainly include case studies (Table 1 and Table 2), owing to the current lack of randomized control trials and the fact that kidney-transplanted patients are consistently excluded in clinical trials of immune checkpoint inhibitors. According to the available data summarized in Table 1, 11 renal transplant patients with advanced cancer were reported to have graft failure after anti-PD-1 treatment. The occurrence of graft failure is mainly caused by acute rejection; however, 4 out of 11 renal transplant patients had graft failure after PD-1 inhibitor treatment without tissue evidence [16,17,18,19]. Acute rejection of transplanted kidney after PD-1 inhibitors occurs mainly through T-cell mediated rejection [20,21,22,23,24,25], although antibody-related rejection [21,22] or vascular rejection [26] has also been reported. The occurrence of acute T-cell-mediated rejection after administration of PD-1 inhibitors may be understood from the viewpoint that the activation of T-cells against donor allograft antigens leads to graft failure via T-cell infiltration of the renal interstitium, renal tubular epithelia, and endothelia. This finding is not surprising because acute interstitial nephritis with infiltration of T-cells and granulocytes in renal tissue after treatment with immune checkpoint inhibitors for malignancies has been reported in non-transplanted patients [27,28]. Acute antibody-mediated rejection may be attributed to the proliferative response of B-cells induced by activated T-cells or activation of memory B-cells expressing PD-1 induced by the concomitant reduction in immunosuppressant use during PD-1 inhibitor treatment [21]. Vascular rejection is mainly caused by cell-mediated rejection; however, antibody-mediated rejection or vascular isolated lesions can contribute to vascular rejection [29]. Graft failure in renal transplant patients usually appears after the first dose of a PD-1 inhibitor which causing a severe graft rejection. However, three renal transplant patients with acute rejection appeared after the second, third, or ninth administration. [22,24,25]. After graft failure, almost all kidneys did not regain function, even after treatment with a high-dose steroid, and patients require hemodialysis for rescue [16,17,20,21,22,23]. Nevertheless, a 64-year-old man with advanced Non-small cell lung cancer (NSCLC) developed graft failure after the ninth cycle of nivolumab, but he did not require hemodialysis for rescue because his graft function improved after high-dose administration of a steroid and increased dosage of mycophenolate mofetil (MMF) and tacrolimus [24]. 

With regard to immunosuppressive regimens before the initiation of a PD-1 inhibitor, five of 11 renal transplant patients with graft failure received only prednisolone monotherapy [16,20,21,23,26]. Two patients had decreased dosage of immunosuppressive medications [22,24]. In one patient, the dosage of immunosuppressive medications was decreased, and tacrolimus was switched to everolimus [25]. In one patient, tacrolimus and MMF were replaced with azathioprine and everolimus [17]. One patient had no immunosuppressive medication [18], and another patient had no information on the titration of immunosuppressive medications [19]. Unsurprisingly, lowering the dose of immunosuppressants before a PD-1 inhibitor significantly increases the risk of graft failure because immunosuppressive therapies are vital in regulating acute allograft rejection and inducing long-term transplanted kidney survival [30].

In terms of the efficacy of PD-1 inhibitors in renal transplant patients with graft failure and advanced cancer, 5 out of the 11 reported patients experienced tumor progression, 4 had a partial response, 1 had a complete response, and 1 had no tumor response data. In three patients with graft failure and advanced cutaneous squamous cell carcinoma (cSCC), all patients exhibited partial and complete response after PD-1 inhibitor treatment. For the renal transplant patient with a complete response of advanced cSCC, he received a combined anti-PD-1 and anti-CTLA4 immunotherapy. However, the patient had sudden cardiac death with unclear etiology during dialysis.

## 4. Intact Graft and No Tumor Progression After a PD-1 Inhibitor in Renal Transplant Patients with Advanced Cancer

Intriguingly, not all renal transplant patients with advanced malignancy experience graft failure after treatment of PD-1 inhibitors. Thus, these cases without graft failure are discussed in this section to provide possible explanations (Table 1). First, a 77-year-old male kidney transplant recipient maintained his graft function after three doses of anti-CTLA-4 antibodies of ipilimumab at a dose of 3 mg/kg every 3 weeks followed by the anti-PD-1 antibody of nivolumab at a dose of 3 mg/kg every 2 weeks for metastatic melanoma. In contrast with other patients with graft failure after ipilimumab followed by PD-1 inhibitors [20,21], this patient maintained an immunosuppressive regimen with 5 mg of prednisolone daily and 5 mg of tacrolimus twice daily before nivolumab [31]. Clinically, the patient’s metastatic melanoma even progressed after nivolumab. Hence, whether the continuation of immunosuppressants reduces the anti-tumor response of PD-1 inhibitors should be further investigated. 

In a second case, a 70-year-old male kidney transplant recipient was treated with nivolumab at a dose of 3 mg/kg every 2 weeks for metastatic duodenal adenocarcinoma after poor response to standard chemotherapy. Immunosuppressive regimens included concurrent prednisolone and the mammalian target of rapamycin (mTOR) inhibitors with sirolimus. Prednisolone was titrated as follows: 40 mg daily 1 week before nivolumab, 20 mg daily after nivolumab, 10 mg daily between 2 weeks and 6 months after nivolumab, and, finally, 5 mg daily. Tacrolimus was replaced by sirolimus before anti-PD-1, and serum sirolimus levels were initially maintained at 4–6 ng/mL after anti-PD-1 and then increased to 10–12 ng/mL 2 weeks after. The patient maintained his graft function without tumor progression [32].

In the third case, a 68-year-old woman with a living-related kidney transplant developed metastatic cSCC. Nivolumab was administrated owing to tumor progression even after radiotherapy, chemotherapy, and targeted therapy. Immunosuppressive regimens included 5 mg of prednisolone daily and sirolimus before initiation of nivolumab. Her tacrolimus was switched early to sirolimus before radiotherapy. The patient’s graft function was well maintained, and no evidence of tumor progression was found after 11 cycles of nivolumab [36]. Calcineurin inhibitor (CNI) minimization or elimination is a critical strategy to decrease CNI toxicities, such as nephrotoxicity, the worsening risk of cardiovascular disease, new-onset diabetes, increased incidence of neoplasms, and viral infections. Some studies have demonstrated well-maintained graft functions without increased rates of graft rejection and failure after CNI elimination using sirolimus in kidney transplantation [37,38]. In addition, the mTOR signaling pathway plays a vital role in tumor initiation and progression. Treatment with mTOR inhibitors can reduce high mTOR signaling levels in various cancer types [39]. Recent studies also suggest that early conversion to an mTOR inhibitor-based maintenance regimen can reduce cSCC [40,41]. Treatment with mTOR inhibitors and concomitant immune checkpoint inhibitors could maintain T-cell energy [42], and mTOR inhibitors have been demonstrated to stimulate naïve T-cell differentiation into Tregs, especially in the presence of IL-2 [33]. The antitumor effect and immunologic tolerance of mTOR inhibitors in renal transplant patients after PD-1 inhibitors must be further investigated. 

In the fourth case, we previously reported a 61-year-old woman who had undergone deceased donor transplantation and eventually advanced urothelial carcinoma. Anti-PD-1 monoclonal antibody (pembrolizumab; 1 mg/kg), humanized anti-vascular endothelial growth factor (VEGF) monoclonal antibody (bevacizumab, 4 mg/kg), and chemotherapy with cisplatin (50 mg/m^2^) and gemcitabine (500 mg/m^2^) were administered intravenously every 3 weeks for 11 cycles. We maintained immunosuppressive regimens, including a fixed dose of mycophenolate mofetil (1 g/day) and 9–10 g/day tacrolimus, to maintain serum tacrolimus levels between 5 and 10 ng/mL before the first dose of pembrolizumab. The patient’s graft function remained stable, and serial images demonstrated significant tumor regression [43]. Platinum-based drugs may enhance the anti-tumor effects of immunotherapy by eliminating immunosuppressive cells, such as Tregs; anti-angiogenic agents may also improve endogenous immune antitumor responses by normalizing the tumor neovasculature [44]. Additionally, acute renal allograft rejection was identified to be associated with increased levels of serum or urine VEGF [45,46]. VEGF inhibitors may prevent graft rejection [47,48], and are currently applied to prevent rejection after corneal transplantation [34,49]. Nevertheless, the humanized anti-VEGF monoclonal antibody should still be investigated in future research in efforts to prevent rejection of transplanted kidney in humans.

According to Tio et al. [19], 4 out of 5 male renal transplant patients with advanced melanoma had intact graft after PD-1 inhibitor treatment. A low rejection rate of renal allograft was observed in this study group after PD-1 inhibitor treatment. These four patients with intact graft were all treated with a PD-1 inhibitor (pembrolizumab), and one of them was treated initially with pembrolizumab followed by an anti-CTLA4 monoclonal antibody with ipilimumab. With regard to the use of immunosuppressive medications, these four patients had immunosuppressive medications before treatment with the PD-1 inhibitor. However, the authors did not mention whether immunosuppressive regimens were continued without titration before the initiation of PD-1 inhibitor treatment. Moreover, concurrent administration of immunosuppressive regimens, including prednisolone, MMF, and mTOR inhibitor (everolimus), was used in only one renal transplant recipient. Whether the initiation of CNI elimination was due to the use of mTOR inhibitor is not clear. Therefore, reviewing the regimen and dosage of immunosuppressive medications before and after PD-1 inhibitor treatment in these four renal transplant patients and exploring why these patients with advanced melanoma were able to maintain graft function after PD-1 inhibitor treatment are necessary. Regarding the outcome of advanced melanoma after pembrolizumab, three out of four renal transplant recipients with intact graft had disease progression, and one had a partial response.

Winkler et al. [35] safely administered anti-PD-1 antibodies to two renal transplant patients with advanced melanoma. One patient was a 60-year-old female renal transplant patient with advanced melanoma treated with four cycles of nivolumab. Her renal function was well maintained after PD-1 inhibitor treatment. Her immunosuppressive regimens included prednisolone and MMF before the PD-1 inhibitor treatment. Cyclosporine was stopped after she was diagnosed with metastatic disease. The other patient was a 55-year-old male renal transplant patient with advanced uveal melanoma treated with four doses of pembrolizumab. His graft function remained stable with mild proteinuria after PD-1 inhibitor treatment. His immunosuppressive medications with cyclosporine and prednisolone were discontinued after diagnosis of uveal melanoma 2 years before anti-PD-1 treatment. The immunosuppressive regimens in the male patient with advanced uveal melanoma remained unchanged after initiation of anti-PD-1 treatment, but the other patient stopped cyclosporine. Nevertheless, the tumors in these two patients progressed even after PD-1 inhibitor treatment. 

In another report by Zehou et al. [50], a 74-year-old male renal transplant patient with advanced melanoma was treated with three cycles of ipilimumab followed by nivolumab at 3 mg/kg, combined with radiotherapy, every 2 weeks. His renal functions initially deteriorated, owing to *Escherichia coli* sepsis, but improved after treatment. The patient remained on immunosuppressants with 5 mg of prednisone, azathioprine, and everolimus before the administration of PD-1 inhibitor. No graft rejection was found. The immunosuppressive regimen of the patient remained unchanged. However, the advanced melanoma continued to deteriorate, and the patient ultimately died 1 year later.

Collectively, the tumor in four out of the 11 renal transplant patients with intact graft responded well to a PD-1 inhibitor. The types of tumor were advanced duodenal adenocarcinoma, advanced cSCC, advanced urothelial carcinoma, and advanced melanoma. Advanced melanoma progressed even after PD-1 inhibitor treatment in seven other renal transplant patients with intact graft. In the report by Tio et al., five out of seven renal transplant patients with intact grafts continued immunosuppressant therapy without titration, and three had immunosuppressive medications, including an mTOR inhibitor.

## 5. Kidney Transplant Patients after PD-1 Inhibitors

Among the 22 renal transplant patients after PD-1 inhibitor treatment, 14 patients developed melanoma (13 cutaneous and one uveal), four developed cutaneous cSCC, two developed NSCLC, one developed a duodenal adenocarcinoma, and one developed urothelial carcinoma. For advanced melanoma in renal transplant patients, the disease control rate with a PD-1 inhibitor was 21%. The disease control rate in renal transplant patients with advanced cSCC was 100%. The patients with duodenal adenocarcinoma and urothelial carcinoma both had a partial response with a PD-1 inhibitor. Eleven out of 22 renal transplant patients (50%) experienced rejection after PD-1 inhibitor treatment. In terms of drug choice of PD-1 inhibitors on renal transplant patients with advanced cancer, 13 patients were administrated with nivolumab and nine patients were administrated with pembrolizumab. 8 out of 13 (61.5%) renal transplant patients with advanced cancer treated with nivolumab had graft failure, whereas 3 out of 9 (33%) renal transplant patients with advanced cancer treated with pembrolizumab had graft failure. Disease control rate in renal transplant patients with advanced cancer administrated by nivolumab and pembrolizumab is 50% and 33 %, respectively. It is difficult to draw conclusions that nivolumab had higher rejection and response rate than pembrolizumab in renal transplant population owing to lack of controlled trial and only a few available cases studies.

## 6. Conclusions

In our review of published cases, PD-1 inhibitors showed anti-tumor effects on advanced malignancies, including metastatic melanoma, cSCC, urothelial tumors, and duodenal adenocarcinoma in renal transplant patients. Interestingly, a high response rate of cSCC and a low response rate of advanced melanoma after PD-1 inhibitor treatment were noted in renal transplant patients. Moreover, PD-1 inhibitors showed a high risk of severe graft rejection without regaining renal function even after treatment with high-dose steroids. Almost all affected renal transplant patients required hemodialysis for rescue. These patients received low-dose or reduced immunosuppressive medications before the initiation of PD-1 inhibitor treatment. By contrast, most renal transplant patients with intact graft continued to take immunosuppressive medications or combined treatment with mTOR inhibitor. For patients who benefited from anti-tumor treatment with anti-PD-1 inhibitors without rejection, two renal transplant recipients were possibly due to the use of low-dose steroid and an mTOR inhibitor, one patient’s advanced urothelial carcinoma regressed after combined treatment with anti-PD-1, anti-VEGF, and chemotherapy without immunosuppressant titration, and one patient had no information in the published article. mTOR inhibitor and anti-VEGF medication may be a key feature for regulating immune tolerance after the administration of PD-1 inhibitors in renal transplant patients. On the basis of these case studies, drawing conclusions regarding the ideal combination of drugs that facilitate anti-PD-1 treatment to achieve the optimal therapeutic effects, and to maintain graft tolerance in renal transplant patients, is difficult. Hence, further studies in renal transplant patients after PD-1 inhibitor treatment are warranted to establish the best treatment strategy and explore an ideal predictive biomarker.

## Figures and Tables

**Table 1:** Clinical response and graft rejection after PD-1 inhibitors in various advanced malignancies of renal transplant patients.

Authors	Year	Types of Advanced Malignancy	Age	Sex	Transplant to Malignancy/CPI (Years)	PD-1 Inhibitors	Concurrent Anti-Cancer Treatment	Immuno-Suppressants	Graft Integrity	Biopsy	Time till Graft Rejection	Rescue	Cancer Outcome
Spain et al. [19]	2016	Melanoma	48	M	12/14	Ipilimumab // nivolumab	Monotherapy	Prednisolone	Rejected	Acute cellular rejection	8 days after 1st nivolumab	HD	PD
Alhamad et al. [20]	2016	Melanoma	68	M	5/6	Ipilimumab // pembrolizumab	Monotherapy	Prednisolone	Rejected	Acute cellular & antibody-mediated rejection	3 weeks after 1st pembrolizumab	HD	PD
Boils et al. [21]	2016	NSCLC	74	M	5/15	Nivolumab	Monotherapy	Prednisolone & cyclosporine	Rejected	Acute cellular & antibody-mediated rejection	3rd nivolumab	HD	No info.
Lipson et al. [22]	2016	cSCC	57	F	5/8	Pembrolizumab	Monotherapy	Prednisolone	Rejected	Acute & chronic cellular rejection	2 months after 1st pembrolizumab	HD	PR
Ong et al. [15]	2016	Melanoma	63	F	3/UK	Nivolumab	Monotherapy	Prednisolone	Rejected	None	1 week after 1st nivolumab	HD	PR
Tamain et al. [23]	2016	NSCLC	64	M	25/UK	Nivolumab	Monotherapy	Tacrolimus & MMF	Rejected	Acute cellular rejection	9th nivolumab cycle	Immuno-suppressants *	PD
Kwatra et al. [16]	2017	Melanoma	58	M	11/11	Pembrolizumab	Monotherapy	Azathioprine & everolimus	Rejected	None	2nd pembrolizumab	Hospice	PD
Miller et al. [17]	2017	cSCC	68	M	6/7	Nivolumab & ipilimumab	Combined	None	Rejected	None	8 days after 1st dual immunotherapy	HD	CR
Deltombe et al. [24]	2017	Melanoma	60	F	11/13	Nivolumab	Monotherapy	Everolimus	Rejected	Acute cellular rejection	25 days after 2nd nivolumab	HD	PD
Goldman et al. [25]	2018	cSCC	50	M	13/13	Nivolumab	Monotherapy	Prednisolone	Rejected	Acute & chronic vascular rejection	13 days after 1st nivolumab	HD	PR
Tio et al. [18]	2018	Melanoma	48	M	0.5/4	Nivolumab	Monotherapy	Prednisone & tacrolimus	Rejected	None	1st nivolumab	HD	PR

Note: NSCLC, non-small cell lung cancer; cSCC, cutaneous squamous cell carcinoma; UK, unknown; NS, not specified; CPI, checkpoint inhibitor; //, followed by, MMF, mycophenolate mofetil; HD, hemodialysis; PD, progressive disease; PR, partial response; CR, complete response; *, renal function improved after methylprednisolone administration and increased dose of MMF and tacrolimus.

**Table 2:** Clinical response and intact graft after PD-1 inhibitors in various advanced malignancies of renal transplant patients.

Authors	Year	Types of Advanced Malignancy	Age	Sex	Transplant to Malignancy/CPI (Years)	PD-1 Inhibitors	Concurrent Anti-Cancer Treatment	Immuno-Suppressants	Graft Integrity	Biopsy	Time till Graft Rejection	Rescue	Cancer Outcome
Herz et al. [30]	2016	Melanoma	77	M	1/8	Ipilimumab // nivolumab	Monotherapy	Prednisone & tacrolimus	Intact	N/A	N/A	N/A	PD
Barnett et al. [31]	2017	Duodenal adenocarcinoma	70	M	5/6	Nivolumab	Monotherapy	Prednisone & sirolimus	Intact	N/A	N/A	N/A	PR
Kittai et al. [32]	2017	cSCC	69	F	4/15	Nivolumab	Monotherapy	Prednisone & sirolimus	Intact	N/A	N/A	N/A	SD
Wu et al. [33]	2017	UC	61	F	5/8	Pembrolizumab	Bevacizumab, cisplatin & gemcitabine	MMF & tarcolimus	Intact	N/A	N/A	N/A	PR
Tio et al. [18]	2018	Melanoma	65	M	NS	Pembrolizumab // ipilimumab	Monotherapy	Prednisone, MMF & everolimus	Intact	N/A	N/A	N/A	PD
Tio et al. [18]	2018	Melanoma	70	M	NS	Pembrolizumab	Monotherapy	Prednisone & tacrolimus	Intact	N/A	N/A	N/A	PD
Tio et al. [18]	2018	Melanoma	75	M	NS	Pembrolizumab	Monotherapy	Prednisone	Intact	N/A	N/A	N/A	PR
Tio et al. [18]	2018	Melanoma	65	M	NS	Pembrolizumab	Monotherapy	Prednisone, MMF & tarcolimus	Intact	N/A	N/A	N/A	PD
Winkler et al. [34]	2018	Melanoma	60	F	11/13	Nivolumab	Monotherapy	Prednisolone & MMF	Intact	N/A	N/A	N/A	PD
Winkler et al. [34]	2018	Melanoma (uveal)	58	M	21/23	Pembrolizumab	Montoherapy	Cyclosporine	Intact	N/A	N/A	N/A	PD
Zehou et al. [35]	2018	Melanoma	74	M	0.5/4	Ipilimumab // nivolizumab	Monotherapy	Prednisolone, MMF & everolimus	Intact	N/A	N/A	N/A	PD

Note: cSCC, cutaneous squamous cell carcinoma; UC, urothelial carcinoma; CPI, checkpoint inhibitor; NS, not specified; //, followed by; MMF, mycophenolate mofetil; N/A, not available; PD, progressive disease; PR, partial response; SD, stable disease.

## Abbreviations

CNI	Calcineurin inhibitor
cSCC	Cutaneous squamous cell carcinoma
CTLA-4	Cytotoxic T-lymphocyte-associated antigen 4
mTOR	Mammalian target of rapamycin
NSCLC	Non-small cell lung cancer
PD-1	Programmed cell death 1
PD-L1	Programmed cell death 1 ligand
SCC	Squamous cell carcinoma
Tregs	Regulatory T-cells
VEGF	Vascular endothelial growth factor
